# Versatile hiPSC Models and Bioengineering Platforms for Investigation of Atrial Fibrosis and Fibrillation

**DOI:** 10.3390/cells15020187

**Published:** 2026-01-20

**Authors:** Behnam Panahi, Saif Dababneh, Saba Fadaei, Hosna Babini, Sanjana Singh, Maksymilian Prondzynski, Mohsen Akbari, Peter H. Backx, Jason G. Andrade, Robert A. Rose, Glen F. Tibbits

**Affiliations:** 1 School of Biomedical Engineering, University of British Columbia, Vancouver, BC V6T 1Z3, Canadakamill_prondzynski@sfu.ca (M.P.);; 2 Cellular and Regenerative Medicine Centre, BC Children’s Hospital Research Institute, Vancouver, BC V5Z 4H4, Canadasaba_fadaei@sfu.ca (S.F.);; 3 Department of Cellular and Physiological Sciences, Faculty of Medicine, University of British Columbia, Vancouver, BC V6T 1Z3, Canada; 4 Biomedical Physiology and Kinesiology, Simon Fraser University, Burnaby, BC V5A 1S6, Canada; 5 Laboratory for Innovations in Microengineering (LiME), Department of Mechanical Engineering, University of Victoria, Victoria, BC V8P 5C2, Canada; 6 Department of Biology, York University, 4700 Keele Street, Toronto, ON M3J 1P3, Canada; 7 Department of Medicine, Montreal Heart Institute, Université de Montréal, Montreal, QC H1T 1C8, Canada; 8 Center for Cardiovascular Innovation, Vancouver General Hospital, Vancouver, BC V5Z 1M9, Canada; 9 Libin Cardiovascular Institute, Cumming School of Medicine, University of Calgary, GAC66, Health Research Innovation Centre, 3280 Hospital Drive NW, Calgary, AB T2N 4Z6, Canada; 10 Department of Molecular Biology and Biochemistry, Simon Fraser University, Burnaby, BC V5A 1S6, Canada

**Keywords:** atrial fibrillation, atrial fibrosis, human-induced pluripotent stem cells (hiPSCs), tissue engineering, 3D bioprinting, disease modeling

## Abstract

**Highlights:**

**What are the main findings?**

- This review identifies a critical gap: existing atrial fibrillation (AF) models (animal, 2D) fail to replicate the complex, 3D interplays between human atrial cells and the fibrotic extracellular matrix.
- We present a conceptual roadmap to address this gap by integrating human-induced pluripotent stem cell (hiPSC)-derived atrial cardiomyocytes and fibroblasts with 3D bioengineering techniques to build functional, human-specific models of atrial fibrosis.

**What are the implications of the main findings?**

- The bioengineered models developed from this roadmap will allow researchers to mechanistically connect specific fibrotic patterns to the arrhythmogenic electrical conduction changes that drive AF.
- These high-fidelity in vitro platforms will accelerate the discovery of novel anti-fibrotic drugs and enable patient-specific testing to advance personalized medicine for atrial fibrillation.

**Abstract:**

Atrial fibrillation (AF) is the most common sustained heart rhythm disorder. It is estimated that AF affects over 52 million people worldwide, with its prevalence expected to double in the next four decades. AF significantly increases the risk of stroke and heart failure, contributing to 340,000 excess deaths annually. Beyond these life-threatening complications, AF results in limitations in physical, emotional, and social well-being causing significant reductions in quality of life and resulting in 8.4 million disability-adjusted life-years per year, highlighting the wide-ranging impact of AF on public health. Moreover, AF is increasingly recognized for its association with cognitive decline and dementia. AF is a chronic and progressive disease characterized by rapid and erratic electrical activity in the atria, often in association with structural changes in the heart tissue. AF is often initiated by triggered activity, often from ectopic foci in the pulmonary veins. These triggered impulses may initiate AF via: (1) sustained rapid firing with secondary disorganization into fibrillatory waves, or (2) by triggering micro re-entrant circuits around the pulmonary venous-LA junction and within the atrial body. In each instance, AF perpetuation necessitates the presence of a vulnerable atrial substrate, which perpetuates and stabilizes re-entrant circuits through a combination of slowed and heterogeneous conduction, as well as functional conduction abnormalities (e.g., fibrosis disrupting tissue integrity, and abnormalities in the intercalated disks disrupting effective cell-to-cell coupling). The re-entry wavelength, determined by conduction velocity and refractory period, is shortened by slowed conduction, favoring AF maintenance. One major factor contributing to these changes is the disruption of the extracellular matrix (ECM), which is induced by atrial fibrosis. Fibrosis-driven disruption of the ECM, especially in the heart and blood vessels, is commonly caused by conditions such as aging, hypertension, diabetes, smoking, and chronic inflammatory or autoimmune diseases. These factors lead to excessive collagen and protein deposition by activated fibroblasts (i.e., myofibroblasts), resulting in increased tissue stiffness, maladaptive remodeling, and impaired organ function. Fibrosis typically occurs when cardiac fibroblasts are activated to myofibroblasts, resulting in the deposition of excessive collagen and other proteins. This change in ECM interferes with the normal electrical function of the heart by creating irregular, fibrotic regions. AF and atrial fibrosis have a reciprocal relationship: AF promotes fibrosis through fibroblast activation and extracellular matrix buildup, while atrial fibrosis can sustain and perpetuate AF, contributing to higher rates of AF recurrence after treatments such as catheter ablation or cardioversion.

## 1. Introduction: Atrial Fibrillation and ECM Remodeling

Atrial fibrillation (AF) is the most common sustained heart rhythm disorder. It is estimated that AF affects over 52 million people worldwide, with its prevalence expected to double in the next four decades. AF significantly increases the risk of stroke and heart failure, contributing to 340,000 excess deaths annually [1,2]. Beyond these life-threatening complications, AF results in limitations in physical, emotional, and social well-being, causing significant reductions in quality of life and resulting in 8.4 million disability-adjusted life-years per year, highlighting the wide-ranging impact of AF on public health [3]. Moreover, AF is increasingly recognized for its association with cognitive decline and dementia.

AF is a chronic and progressive disease characterized by rapid and erratic electrical activity in the atria, often in association with structural changes in the heart tissue [4]. AF is frequently initiated by triggered activity originating from ectopic foci in the pulmonary veins, driven by abnormal automaticity and afterdepolarizations. These triggered impulses may initiate AF via (1) sustained rapid firing with secondary disorganization into fibrillatory waves, or (2) by triggering micro re-entrant circuits around the pulmonary venous-LA junction and within the atrial body. In each instance, AF perpetuation requires a vulnerable atrial substrate that stabilizes re-entrant circuits through slowed and heterogeneous conduction and functional conduction abnormalities (e.g., fibrosis, disrupted intercalated disk structure, and impaired Na^+^ channel function), all of which promote reduced conduction velocity and heterogeneity [5]. The re-entry wavelength, determined by the product of the conduction velocity and refractory period, is shortened by slowed conduction, favoring AF maintenance.

One major factor contributing to these changes is the disruption of the extracellular matrix (ECM), which is induced by atrial fibrosis. Fibrosis-driven disruption of the ECM, especially in the heart and blood vessels, is commonly caused by conditions such as aging, hypertension, diabetes, smoking, and chronic inflammatory or autoimmune diseases [6,7,8]. These factors lead to excessive collagen and protein deposition by activated fibroblasts (i.e., myofibroblasts), resulting in increased tissue stiffness, maladaptive remodeling, and impaired organ function. Fibrosis typically occurs when cardiac fibroblasts are activated to myofibroblasts, resulting in the deposition of excessive collagen and other proteins. This change in ECM interferes with the normal electrical function of the heart by creating irregular, fibrotic regions [9].

AF and atrial fibrosis have a reciprocal relationship: AF promotes fibrosis through fibroblast activation and ECM buildup, while atrial fibrosis can sustain and perpetuate AF, contributing to higher rates of AF recurrence after treatments such as catheter ablation or cardioversion [10].

The atrial myocardium’s cellular composition underscores the importance of the ECM. Although cardiomyocytes (CMs) account for ~75% of the myocardial volume, they constitute a significantly lower percentage of cells in the atrial tissue. There is some controversy about the proportion of non-myocytes (e.g., fibroblasts, endothelial cells) compared to the number of cardiomyocytes [11]. Reported estimates for the proportion of cardiac fibroblasts (CFs) relative to CMs vary widely, with some studies suggesting a CF:CM ratio as high as 4:1, while others report much lower ratios, such as 1:10 [12,13,14]. This variability likely reflects several factors. First, the ratio of fibroblasts varies between species. Second, within the same species the ratios of fibroblasts to cardiomyocytes vary within different regions of the same heart, and with developmental stage, as embryonic, neonatal, and adult hearts each possess distinct cellular compositions. Third, methodological differences between studies further contribute to the discrepancies in reported ratios; with techniques such as flow cytometry, immunohistochemistry, and single-cell RNA sequencing providing divergent results depending on the markers selected and the protocols used for cell isolation. Finally, the defining features of fibroblasts remain complex due to the limited specificity of many commonly used markers. For example, proteins such as DDR2, vimentin, and α-SMA are expressed in fibroblasts but also overlap with other mesenchymal populations, including pericytes and vascular smooth muscle cells. As a result, studies relying on these markers may inadvertently include non-fibroblast populations, contributing to variability in reported ratios [15,16].

Despite these discrepancies, the consensus is that fibroblasts constitute a substantial and functionally critical component of the myocardium, influencing both the ECM and the electrophysiological and mechanical environment. Recognizing the uncertainty in precise cell ratios is important when interpreting experimental data and modeling cardiac tissue in vitro. Engineered tissue models, for instance, must carefully consider which ratios to employ, as these can significantly impact tissue structure, function, and disease modeling outcomes.

Fibroblasts synthesize and secrete the ECM network that surrounds myocytes. In normal physiology, fibroblasts and ECM proteins (e.g., collagen) electrically insulate muscle bundles and transmit mechanical forces. In the healthy heart, cardiac fibroblasts physically interact with adjacent cardiomyocytes, forming close membrane contacts that are thought to contribute to the structural integrity and mechanical function of the myocardium. These interactions may also influence cardiomyocyte behavior through both direct cell-cell contact and paracrine signaling, supporting proper alignment, electrophysiological stability, and metabolic homeostasis. Importantly, while both modes of fibroblast–cardiomyocyte communication are recognized, their relative contributions remain unclear. Direct physical contact, such as adherens junctions and gap junctions, may allow fibroblasts to mechanically stabilize the myocardium and even electrically couple with cardiomyocytes, influencing conduction properties. In parallel, fibroblasts secrete a wide range of paracrine factors—growth factors, cytokines, and extracellular vesicles—that can modulate cardiomyocyte hypertrophy, metabolism, and excitability. In AF, both mechanisms are thought to play roles: gap-junctional coupling could create conduction heterogeneity and arrhythmogenic substrates, while paracrine signals could promote structural remodeling and metabolic dysregulation. However, because these processes often occur simultaneously and can influence each other, it has been difficult to determine which mechanism is dominant under different pathological conditions. This uncertainty highlights a significant gap in AF research and underscores the need for studies that disentangle the effects of direct contact versus secreted signaling in driving arrhythmogenesis.

Such fibroblast-cardiomyocyte contact has been confirmed in both in vivo and in vitro settings and is increasingly recognized as a key component of myocardial tissue organization and function. In pathology, activated fibroblasts (i.e., myofibroblasts) secrete excessive amounts of collagen and other ECM proteins, driving fibrosis that disrupts tissue organization and contributes directly to AF. In addition, myofibroblasts upregulate gap junction proteins (e.g., connexins) that allow direct electrical coupling with cardiomyocytes, altering local conduction properties. These processes occur alongside the progressive deposition of collagen, which separates cardiomyocyte bundles and further exacerbates conduction heterogeneity [17].

The intricate crosstalk between cardiomyocytes and fibroblasts, coupled with the pathological deposition of the extracellular matrix, creates a complex fibrotic substrate that underpins the progression of AF. Understanding and targeting this substrate is paramount, yet current models have significant limitations in their ability to recapitulate the necessary human-specific cellular and structural complexities. To address this critical gap, the central purpose of this review is to provide a conceptual roadmap for engineering and validating human-induced pluripotent stem cell (hiPSC)-based models of atrial fibrosis to study AF. This roadmap will first establish the need for this bioengineered approach by outlining the limitations of existing preclinical and clinical methods. It will then detail the essential components, from generating patient-specific atrial cardiomyocytes and fibroblasts to developing biomimetic bioinks for advanced 3D bioprinting techniques. Finally, we will propose a multi-step framework for building, inducing pathology within, and characterizing these engineered tissues to ensure they can yield meaningful pathophysiological insights and serve as a robust platform for accelerating the development of targeted, patient-specific therapies.

## 2. Limitations of Current AF Models and Therapies

Each model used to study AF—whether human or animal model-based, primary cells, or hiPSC-derived—has its own advantages and limitations. A balanced, integrative approach is needed to leverage their respective strengths for studying atrial fibrosis.

### 2.1. Animal Models

Small animal models, such as mice, offer distinct advantages, as an intact organism can play a complex role in the development of fibrosis. Using whole organisms makes it possible to assess not only local cellular processes but also systemic factors, including neurohumoral factors, that cannot be adequately modeled. Crucially, intact animal models enable researchers to study the recruitment of circulating cells, including immune cells and potentially bone marrow-derived progenitor cells, to sites of injury. This systemic response, which has been implicated in human AF pathophysiology [18], involves the dynamic interplay between immune cells and cardiac fibroblasts during fibroblast activation, and the subsequent contributions to myocardial remodeling. These models also allow examination of other native tissue processes, such as vascular responses, electrical conduction, and the development of arrhythmogenic conduction patterns in the heart, which are highly dependent on the heart’s three-dimensional architecture. Thus, whole-animal approaches are essential for understanding how fibrosis progresses in the context of complex cell–cell and tissue–environment interactions, ultimately bridging the gap between molecular findings and clinically relevant outcomes.

Like all models, mouse models also have some disadvantages. In this case, they are limited in their ability to fully replicate human atrial electrophysiology, as the intrinsic beating rate is almost an order of magnitude higher, and they lack several key ion channels involved in human AF. Larger animal models (e.g., canine and porcine) can develop AF and fibrosis when subjected to stressors like rapid pacing, but these models are extremely costly and still differ from humans in meaningful ways [1]. For instance, in these animals, re-entrant arrhythmias often require significant fibrosis, whereas in humans, AF can occur with variable levels of fibrosis, including minimal fibrosis. Additionally, species-specific variations in cardiac anatomy, electrophysiology, conduction system structure, and cellular composition mean that the mechanisms underlying AF and fibrosis differ [19,20]. This emphasizes both the species-specific differences and the crucial role of fibrosis in AF [9].

### 2.2. Ex Vivo and 2D Cell Models

Primary human atrial cardiomyocytes cultured in vitro offer the advantage of precise experimental control, allowing studies of cellular mechanisms under defined conditions, including electrical stimulation to maintain more physiological behavior (e.g., pacing to preserve function) and acute investigation of myocardial responses shortly after isolation [21]. Compared to whole tissue models, isolated cells facilitate the investigation of specific cellular phenotypes without the complexity of systemic influences. Building on this, an increasingly important platform is the use of ex vivo human living myocardial slices, also known as precision-cut cardiac slices (PCCS). This method allows for atrial tissue obtained during surgeries (e.g., right atrial appendage biopsies from patients with AF) to be cultured as thin (e.g., 300–400 µm), viable slices [22]. These slices are a powerful model because they preserve the native three-dimensional architecture, multicellular composition (cardiomyocytes, fibroblasts, etc.), ECM, and cellular connectivity. They can be cultured for several days, enabling more tissue-relevant studies for elucidating disease mechanisms or for drug screening [23] than dissociated cells. This tissue culture is less costly and faster to prepare than ventricular slices and can be derived from more accessible human atrial samples.

However, there are several disadvantages: primary human atrial cardiomyocytes rapidly de-differentiate in culture, losing their mature structural and functional characteristics within days, including changes in sarcomere organization and ion channel expression, which limits their use mostly to short-term studies [24] Traditional 2D cultures on rigid plastic substrates fail to replicate the soft, 3D ECM environment of the heart, causing the unnatural activation of resident fibroblasts and often rendering them overactive, resulting in fibrosis-like ECM patterns that do not faithfully reflect human disease pathology. Moreover, these systems lack realistic ECM and fail to mimic the natural cell-cell and cell-matrix interactions. While atrial tissue slices preserve native architecture and intercellular connectivity better than dissociated cells, their viability and electrical function deteriorate relatively quickly (usually within days to weeks), limiting long-term functional studies or scalability for patient-specific applications [25]. Furthermore, the irregular cardiomyocyte orientation and trabeculated atrial structure complicate preparing consistent tissue slices for culture [25,26]. Additionally, native human atrial tissue samples are almost always obtained from patients with underlying heart disease, making it challenging to find truly healthy controls. Patient-to-patient variability, including factors such as age, sex, and comorbidities, further complicates the interpretation and reproducibility of results.

### 2.3. Clinical Imaging and Surrogates

Late gadolinium enhancement (LGE) MRI and electro-anatomical voltage mapping are routinely used for detecting cardiac fibrosis, but their roles in guiding atrial ablation are increasingly debated. While LGE MRI is validated for ventricular scar imaging, its application in atrial fibrosis is far less robust, recent multicenter trials, including DECAAF-II (the only randomized controlled trial using MRI guidance for ablation), showed no improvement in arrhythmia recurrence when adding MRI-guided fibrosis ablation to pulmonary vein isolation, and also reported a greater risk of procedural complications [27]. The question of whether atrial fibrosis can be imaged with LGE in a clinically meaningful way, contrasting with its proven utility in ventricular scar assessment, remains unclear [28].

Most clinical protocols for “scar-guided” ablation rely on invasive electro-anatomic mapping rather than non-invasive MRI, but evidence supporting this strategy is conflicting, several studies report neutral results, with only isolated positive trials that have not shifted community consensus. Both LGE-MRI and voltage mapping infer fibrosis indirectly and are prone to limited sensitivity for diffuse interstitial fibrosis or quantifying fibroblast activity. Additionally, neither modality enables direct mechanistic interrogation or drug testing on fibrotic tissue, nor do they resolve cellular and molecular interactions involved in disease progression.

Emerging digital twin approaches, where patient-specific imaging data and computational models simulate cardiac function and fibrotic remodeling, may eventually bridge these gaps by providing a more mechanistic, predictive, and individualized strategy for understanding and treating arrhythmogenic substrates [29,30,31]. Digital twin models can incorporate multi-scale data but remain in early phases of clinical validation and are not yet a substitute for empirical mapping and imaging in procedural planning.

### 2.4. Current Therapies

Recent landmark trials have established catheter ablation as a highly effective rhythm-control strategy, with studies demonstrating its superiority over antiarrhythmic drugs as a first-line therapy for select patients with atrial fibrillation [32]. However, both antiarrhythmic drugs and catheter ablation primarily address the electrical aspects of AF and do not reverse the underlying structural remodeling, particularly atrial fibrosis. This limitation contributes to suboptimal long-term outcomes, as approximately 50% of patients with persistent AF experience arrhythmia recurrence within 12 months of an ablation procedure [33,34].

Crucially, the presence and extent of left atrial fibrosis directly impact the reduction in AF burden following catheter ablation [35]. This highlights a significant therapeutic gap, as the fibrotic substrate responsible for maintaining the arrhythmia remains untreated. While emerging anti-fibrotic strategies, such as mineralocorticoid receptor antagonists, have shown promise in reducing atrial fibrosis in preclinical models, their mechanisms in human atria are not fully understood [36]. Therefore, there is a critical need for human-relevant, scalable models that incorporate the ECM and fibroblast components of atrial remodeling to test new therapies and understand AF’s progression.

Choosing an appropriate model to study atrial fibrosis and AF involves navigating a complex landscape of trade-offs between physiological relevance, experimental control, and translational potential. As summarized in Table 1, each existing modality occupies a different niche, with distinct strengths and weaknesses. Critically, no single model perfectly recapitulates all aspects of human AF. While animal models excel at capturing systemic complexity, their divergent electrophysiology fundamentally limits their utility for dissecting human-specific arrhythmia mechanisms. Conversely, 2D cell cultures offer high-throughput capabilities but do so at the cost of profound biological simplification, failing to model the crucial three-dimensional cell-matrix interactions that drive fibrotic remodeling. This analysis reveals a critical gap in the field: the need for a model that combines the human-specific genetics of hiPSCs with the structural and multicellular complexity of native tissue. The 3D bioprinting roadmap proposed in this review aims to directly address this gap, offering a balanced approach to bridge the divide between simplistic in vitro systems and complex but less translatable animal models.

Collectively, the limitations of animal models, the instability of primary cell cultures, the indirect nature of clinical imaging, and the focus of current therapies on electrical symptoms rather than structural causes create a compelling need for a new paradigm. An ideal model would be human-specific, scalable, structurally and functionally controllable, and capable of incorporating the key cellular and matrix players in atrial fibrosis.

## 3. Advances in hiPSC-Derived Cardiac Cells for Modeling AF

The first critical step in our roadmap for engineering a human atrial fibrosis model is the generation of a reliable, and patient-specific supply of the key cardiac cell types. Human-induced pluripotent stem cells (hiPSCs) have emerged as the cornerstone technology for this purpose, overcoming the limitations of scarcity and de-differentiation associated with primary human cells. A major enabling technology for patient-specific cardiac models is human-induced pluripotent stem cells (hiPSCs). HiPSCs can be differentiated into relevant cardiac cell types—notably atrial cardiomyocytes (hiPSC-aCMs), cardiac fibroblasts (hiPSC-CFs), and other cardiac cells (e.g., endothelial cells), providing an unlimited, patient-specific cell supply. Recent advances address previous challenges in obtaining mature, chamber-specific cells.

### 3.1. hiPSC-Derived Atrial Cardiomyocytes (hiPSC-aCMs)

Recent work by Keller and colleagues has demonstrated that atrial and ventricular cardiomyocytes originate from distinct mesodermal populations, which can be differentially specified during early stages of PSC differentiation [43]. Atrial cardiomyocytes are derived from a mesodermal population characterized by the expression of *RALDH2* (retinaldehyde dehydrogenase 2) and *PDGFRα* (platelet-derived growth factor receptor alpha). *RALDH2* is a key enzyme involved in retinoic acid synthesis, and its expression marks mesodermal cells that are competent to respond to retinoic acid signaling, which is crucial for atrial specification. In contrast, ventricular cardiomyocytes originate from a separate mesodermal population marked by *CD235a* (also known as glycophorin A) and *PDGFRα*. These *CD235a*^+^ *PDGFRα*^+^ mesodermal cells are distinct from the *RALDH2*^+^ population and are poised to give rise to ventricular, rather than atrial, cardiomyocytes.

**Figure 1:**
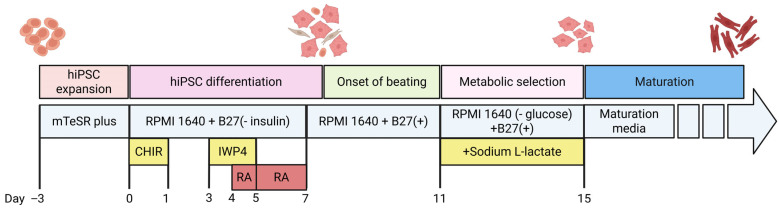
Schematic timeline of hiPSC differentiation into atrial cardiomyocytes, metabolic purification, and maturation. Human-induced pluripotent stem cells (hiPSCs) were expanded in mTeSR Plus medium (Day −3 to 0) and differentiated toward the cardiac lineage using a small-molecule Wnt-modulation protocol. CHIR99021 treatment (Day 0–1) was followed by IWP-4 inhibition (Day 3–4) and retinoic acid (RA) exposure (Day 4–6) to direct atrial specification. Cardiomyocyte beating initiated around Day 7 under RPMI-1640 + B27 (with insulin) maintenance. Metabolic selection was performed from Day 11–15 using glucose-free RPMI + B27 supplemented with sodium L-lactate to enrich cardiomyocytes. Subsequently, cells were cultured in maturation medium to promote structural and functional maturation of atrial cardiomyocytes. Created in BioRender. https://BioRender.com/95lcbg7 (accessed on 4 November 2025).

During directed differentiation (Figure 1), retinoic acid (RA) signaling between days 3–5, corresponding to the mesodermal stage, plays a crucial role in atrial specification [44]. RA acts on *RALDH2*-expressing mesoderm to promote an atrial fate, upregulating atrial markers such as *KCNJ3*, *NPPA*, and *MYL7*, while suppressing ventricular markers like *MYL2* and *IRX4*. Specifically, *KCNJ3* encodes the Kir3.1 potassium channel subunit important for regulating electrical activity in atrial cells; *NPPA* encodes atrial natriuretic peptide, a hormone that helps regulate fluid balance, blood pressure and cardiac structure; *MYL7* encodes atrial myosin light chain, which contributes to atrial muscle contraction. In contrast, *MYL2* encodes a ventricular myosin light chain protein, and *IRX4* is a transcription factor critical for ventricular development and identity. This gene expression pattern induced by RA supports the development of functional atrial cardiomyocytes by promoting atrial electrical and contractile properties while repressing ventricular characteristics [45]. These findings emphasize that the precise timing and patterning of mesoderm, using RA and BMP/Activin gradients is critical for producing enriched populations of chamber-specific cardiomyocytes [46].

However, despite these achievements, hiPSC-aCMs without any intervention are still functionally immature compared to adult atrial cells. They typically resemble fetal cells, with spontaneous activity and slow conduction. To address this, several maturation strategies have been developed, including prolonged culture in structured 3D environments, co-culturing with fibroblasts, electrical stimulation, hormone exposure, and the use of engineered tissues. One recent study found that co-culturing hiPSC-aCMs with fibroblasts in a patterned 3D matrix for over six weeks greatly enhanced their maturity across structural, electrical, and metabolic features [1].

An important advantage of hiPSC-aCMs is that they retain the genetic background of the donor, making them highly valuable for studying genetic causes of AF, such as ion channel variants or titin truncations, in a personalized, controllable model. hiPSC-aCMs have already been used to investigate familial AF and test atrial-specific drugs like vernakalant [1], highlighting their relevance in precision medicine.

### 3.2. hiPSC-Derived Cardiac Fibroblasts (hiPSC-CFs)

**Figure 2:**
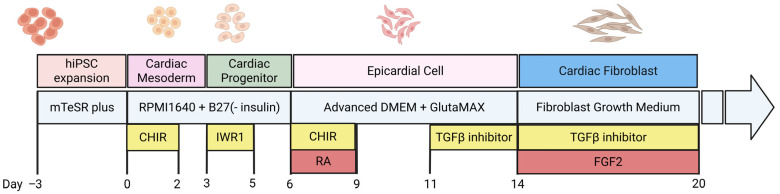
Directed differentiation timeline for generating cardiac fibroblasts from hiPSCs. Human-induced pluripotent stem cells (hiPSCs) were expanded in mTeSR Plus medium (Day −3 to 0) and differentiated via temporal modulation of Wnt signaling to induce mesoderm and cardiac progenitor formation. CHIR99021 was applied at Day 0 to activate Wnt signaling, followed by IWR-1-mediated Wnt inhibition on Days 3–5 to promote cardiac mesoderm and progenitor specification. Cells were transitioned to Advanced DMEM + GlutaMAX on Day 6, and retinoic acid with a second CHIR pulse (Days 6–9) was used to drive epicardial lineage commitment. Epicardial-derived fibroblast induction was achieved with sequential TGF-β inhibition (Day 11–20) and pro-fibrotic stimulation with FGF-2 from Day 14 onward. Cells were maintained in fibroblast growth medium for continued maturation beyond Day 20. Created in BioRender. https://BioRender.com/6zwc46x (accessed on 4 November 2025).

Although cardiomyocytes have received most of the experimental attention, cardiac fibroblasts play a vital role in regulating fibrosis and cardiac tissue behavior. Recent protocols allow for the generation of non-activated cardiac fibroblasts from hiPSCs [47,48], which can be expanded, stored, and used consistently (Figure 2). These fibroblasts are genetically matched to hiPSC-derived cardiomyocytes from the same patient, enabling fully human, patient-specific models.

Similarly, hiPSC-derived cardiac fibroblasts also pass through a mesodermal stage, typically characterized by PDGFRα expression. Their development is influenced by early mesodermal signals, including Wnt, BMP, and TGF-β, and they may arise from either epicardial or lateral plate mesoderm precursors. The differentiation strategy affects not only lineage commitment but also fibroblast plasticity and activation potential, which is crucial for engineering fibrotic cardiac tissues.

It is important to acknowledge that fibroblasts are a highly heterogeneous cell population, both in origin and function. During embryogenesis, cardiac fibroblasts can arise from multiple sources, including the epicardium (via epithelial-to-mesenchymal transition), endocardium, and second heart field mesoderm [49]. This contributes to regional diversity within the heart.

In particular, atrial fibroblasts are increasingly recognized as distinct from ventricular fibroblasts, differing in gene expression profiles, receptor composition, cytokine responsiveness, and electrophysiological interactions with cardiomyocytes [37]. For example, atrial fibroblasts have been shown to express higher levels of pro-fibrotic genes in response to atrial stretch or AF-like stimulation and may secrete different ECM components or paracrine factors compared to their ventricular counterparts.

A key advantage of hiPSC-CFs is that they can be selectively activated. When exposed to fibrotic triggers like TGF-β1, they transition into myofibroblasts, characterized by increased expression of markers including collagen I (COL1A1), α-smooth muscle actin (ACTA2), and, importantly, periostin (POSTN). For instance, Cumberland et al. [50] showed that in the absence of TGF-β, these cells maintain a non-activated state (vimentin-positive, *αSMA*-negative), but upon TGF-β1 stimulation, they exhibit a 3-fold increase in collagen type I and a 5-fold increase in *ACTA2* expression, as detected by αSMA immunostaining, confirming their functional responsiveness [47,50].

By combining hiPSC-CFs with hiPSC-aCMs in 3D engineered tissues, genetically uniform heart models can be created that avoid species mismatch and immune incompatibilities. Co-culturing cardiomyocytes with fibroblasts improves their maturation, leading to slower, more adult-like beating rates and stronger contractions [51]. Fibroblasts also contribute to ECM production and paracrine signaling, promoting better alignment, electrical coordination, and long-term viability. In doing so, they help cardiomyocytes maintain structural and electrical integrity over time [42]. These findings emphasize that multicellular heart models, that include fibroblasts, better replicate real human heart tissue and disease mechanisms than cardiomyocytes alone.

## 4. Generating Atrial Fibrotic Tissue

The next step of the roadmap outlines strategies to assemble these cell types into multicellular tissue constructs using 2D co-culture, 3D engineered tissues, and bioprinting platforms, followed by controlled induction of fibrosis to recapitulate the structural and functional hallmarks of atrial disease.

### 4.1. 2D Monolayer Approach

In vitro cellular models have become increasingly valuable in fibrosis research. Among these, two-dimensional (2D) models, in which cells are cultured in monolayers, offer several distinct advantages, including low cost, ease of high-throughput experiments, and straightforward analytical methods. However, 2D models lack the structural complexity and precise ECM interactions present in native tissues, oversimplifying the complex fibrotic environment and reducing their physiological relevance compared to 3D models. Thus, 2D models are often used alongside other approaches [38].

To create a robust and reproducible 2D model for atrial fibrosis relevant to AF, selecting appropriate cell types is a key factor. The most used cell types are cardiac fibroblasts, followed by their combination with cardiomyocytes (CM) and, in some cases, endothelial cells (EC) [38]. Multicellular systems comprising fibroblasts and cardiomyocytes can better model the effects of anti-fibrotic drugs on fibroblasts, cardiomyocytes, and cell–cell interactions [37]. These cells can be derived from human sources, thereby enhancing the translational relevance of the model, such as human-induced pluripotent stem cell-derived cardiac cells or commercially available human primary cardiac fibroblasts (hCF). The robust differentiation protocols pioneered by Giacomelli et al. [39]. Generating both hiPSC-derived cardiomyocytes and fibroblasts has become foundational for creating reproducible and physiologically relevant cardiac co-cultures. Alternatively, primary cardiac fibroblasts isolated from murine hearts can also be employed. These cells are cultured in defined ratios to establish a controlled co-culture environment. A distinct advantage of using all hiPSC-derived cardiac cells is that it provides a fully human model, which is more relevant for studying human heart diseases. The use of hiPSC-derived cells also allows for the study of specific genetic variants, which have been linked to an increased risk of AF [52].

A ratio of 3:1 (CM to CF) has been used to mimic healthy tissue, and 1:3 to mimic fibrotic tissue [38] although this is controversial. 2D models are typically cultured in monolayers on tissue culture polystyrene (TCP) plates. Since CFs respond to mechanical cues and become activated in substrates of higher stiffness, the surface of TCP plates can be coated with commercial ECM components such as commonly used but costly proteins like fibronectin and laminin that aid growth and differentiation, or complex coatings like Matrigel and Cultrex to minimize uncontrolled CF activation during cell maintenance [40,41]. Culturing cells on soft matrices that mimic the physiological stiffness of cardiac tissue helps maintain cell phenotype and function. Softer substrates have been shown to better support cardiomyocyte contractility and reduce unwanted fibroblast activation, making them beneficial for more accurate modeling of both healthy and fibrotic conditions [53].

Previous studies have demonstrated that incorporating cardiac fibroblasts into cultures of hiPSC-derived cardiomyocytes enhances tissue-like features. For example, Beauchamp et al. and Giacomelli et al. [39,42] showed that co-cultures of hiPSC-CMs with fibroblasts led to improved structural organization, increased ECM deposition, and enhanced contractile function compared to monoculture spheroids. These findings support the idea that fibroblasts play a critical role in cardiac maturation and function, suggesting that even in 2D settings, co-culture systems can better replicate aspects of the native cardiac environment compared to cardiomyocyte-only models. Iseoka et al. [37] developed human iPSC-derived cardiac tissue fibrosis models, showing that co-culture systems can successfully mimic fibrotic remodeling and provide a platform for drug screening. Together, these studies emphasize that cardiomyocyte–fibroblast co-cultures are not only physiologically relevant but also highly versatile for modeling disease processes such as AF.

### 4.2. 3D Bioprinting Technologies

Studies of 3D engineered cardiac tissues co-culturing cardiomyocytes and cardiac fibroblasts have made substantial progress in cardiac tissue engineering by creating models that more closely mimic the physiological environment of the human heart. Alongside 3D spheroids, self-assembling cardiac organoids have also emerged as a powerful platform, capable of recapitulating aspects of early heart development and multicellular complexity, providing a valuable tool for these research questions [54,55]. Incorporating fibroblasts alongside hiPSC-derived cardiomyocytes—in both organoid and other engineered tissue models—significantly improves cardiomyocyte maturation, structural organization, and extracellular matrix production. This co-culture enhances electrical coupling, resulting in stronger contractions, improved alignment, and prolonged tissue viability. Such 3D multicellular models better replicate native heart tissue heterogeneity and disease mechanisms compared to cardiomyocyte-only cultures, making them valuable platforms for studying cardiac biology and pathology [42,56].

**Figure 3:**
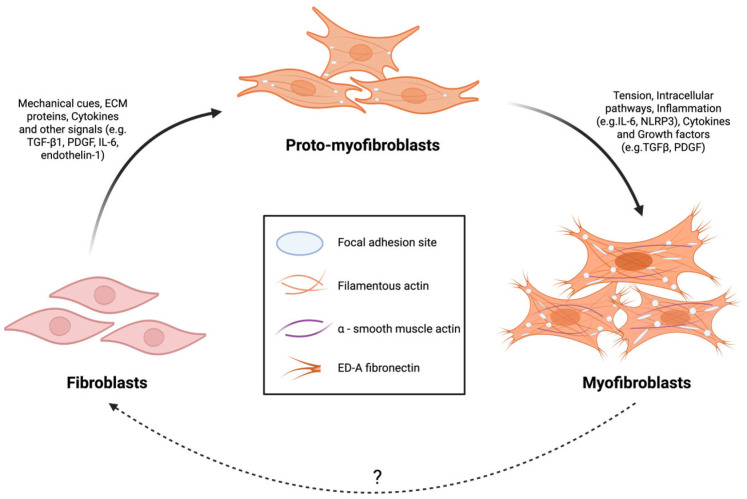
Schematic depiction of fibroblast activation and transition to myofibroblasts. Quiescent fibroblasts respond to mechanical cues, ECM proteins, cytokines, and other profibrotic signals to adopt a proto-myofibroblast phenotype characterized by increased filamentous actin and early expression of ED-A fibronectin and focal adhesion structures. Sustained mechanical tension, intracellular signaling, and inflammatory cytokines further drive differentiation into mature myofibroblasts, which are defined by prominent α-smooth muscle actin (α-SMA) stress fibers, robust focal adhesions, and enhanced ECM deposition. Myofibroblast persistence contributes to pathological fibrosis, while resolution pathways may allow reversion toward a fibroblast-like state. Created in BioRender. https://BioRender.com/bjw3ils (accessed on 4 November 2025).

Recent advances in engineered tissue approaches have demonstrated enhanced anisotropic alignment of cardiomyocytes within 3D microtissues, along with improved ECM deposition and beneficial paracrine signaling from fibroblasts (Figure 3). These features collectively maintain electrical integrity and stable contractility over extended culture periods. The creation of biomimetic multicellular environments that reflect the complex architecture of the myocardium marks a significant milestone in cardiac tissue engineering, particularly for modeling fibrotic conditions [1,57].

Building on these developments, 3D bioprinting offers a next-level capability by enabling precise spatial patterning of multiple cardiac cell types—cardiomyocytes, fibroblasts, and endothelial cells—within ECM-based bioinks. This spatial control is essential for reproducing the microstructural heterogeneity characteristic of native and fibrotic myocardium, such as patchy fibrosis observed in atrial disease. Through controlled placement of cells, bioprinting better mimics tissue anisotropy and fibrotic patchiness, optimizing cell-cell and cell-ECM interactions. Additionally, 3D bioprinting facilitates the creation of perfusable, vascular-like channels within constructs, enhancing nutrient delivery and long-term tissue viability. This combination of multicellular complexity and hierarchical spatial structuring enables the fabrication of cardiac tissue models that are more physiologically and pathologically relevant for mechanistic studies, drug screening, and potentially regenerative therapies [58,59].

#### 4.2.1. Bioinks for Cardiac Tissue

A variety of biomaterials can be used as bioinks in cardiac bioprinting, including natural hydrogels such as alginate, collagen, gelatin, and hyaluronic acid, as well as decellularized ECM extracts. For heart applications, bioinks must be biocompatible, support cell viability, and exhibit soft, elastic properties that closely match those of cardiac tissue. Composite bioinks, such as collagen combined with hyaluronic acid, have been used to encapsulate hiPSC-derived cardiomyocytes for direct 3D printing. Adding heart-specific ECM components (e.g., laminin or decellularized myocardial matrix) enhances cell adhesion and maturation by providing natural biochemical signals [11]. Bioinks can also be tailored to model fibrosis: for example, collagen-rich inks mimic stiff, fibrotic tissue, while softer fibrin-based inks may maintain fibroblasts in a non-activated state [60].

#### 4.2.2. Bioprinting Techniques

Among the various methods, extrusion-based bioprinting is the most common for cardiac constructs. It uses a syringe-like nozzle to deposit thick, cell-rich bioinks layer by layer, supporting high cell densities (~10^8^–10^9^ cells/mL). This approach is suitable for printing both simple heart patches and complex structures. Other techniques, such as inkjet and laser-assisted bioprinting, offer finer resolution by dispensing tiny droplets, but usually support lower cell densities. These are particularly useful for modeling small-scale atrial fibrotic architecture, where precise spatial control is required to replicate fibroblast-rich patches within hundreds of microns. Innovations like Freeform Reversible Embedding of Suspended Hydrogels (FRESH) bioprinting allow high-resolution printing of soft, delicate bioinks by temporarily suspending the structure in a support bath, enabling fabrication of complex 3D constructs without collapse [61].

#### 4.2.3. Vascularization and Maturation

One major challenge in building thick cardiac tissues (>300 µm) is the diffusion of O_2_ and nutrients to the core, particularly at high stimulation frequencies and at higher temperatures (e.g., 37 °C). To address this, printed tissues can include pre-formed channels or sacrificial fibers that develop into vascular networks. For example, sacrificial writing techniques (e.g., co-SWIFT) have been used to pattern hierarchically branching vascular networks within dense cardiac spheroids [62]. Other approaches have successfully printed thick, perfusable cardiac patches by co-printing cell-laden bioinks for the parenchyma with a separate bioink for the blood vessels [63]. Additionally, culturing these constructs in bioreactors with mechanical stretching or electrical stimulation enhances cell alignment, electrophysiology, and maturation. For instance, bioprinted atrial muscle strips can be cyclically paced to mimic native heart activity. This combination of precise 3D patterning, biochemical optimization, and dynamic conditioning enables the creation of cardiac tissues that more closely resemble functional human myocardium compared to traditional methods like casting or spontaneous cell assembly [9].

Overall, 3D bioprinting offers unprecedented control over tissue geometry and composition. In the context of AF and fibrosis, it has the potential to create an in vitro atrial tissue that contains regions of high collagen density and fibroblast concentration adjacent to regions of healthy myocardium, mirroring the heterogeneity observed in a fibrotic atrium [64]. This capability is crucial for studying how patterns of fibrosis and altered cell-cell coupling lead to arrhythmia. It also provides a platform to test interventions (drugs, gene therapies) in a setting where the ECM structure is present and modifiable [59].

## 5. Characterization of In Vitro Atrial Fibrosis Models

Cardiac tissue models have become invaluable for recapitulating key aspects of human cardiac physiology and pathophysiology in vitro. Among emerging applications, the development of fibrotic cardiac tissue models has gained significant traction as a model to study cardiac fibrosis, a hallmark of many cardiovascular diseases, including heart failure and arrhythmogenic cardiomyopathies [65]. Building on the above advancements, one can use a multi-step research program to engineer fibrotic atrial tissue models using hiPSCs, tailored bioinks, and 3D bioprinting. The aim is to recreate key aspects of atrial fibrosis—excessive ECM deposition, fibroblast-myocyte interactions, and the resultant electrophysiological changes—in a controlled setting. The iterative steps are described below.

### 5.1. Bioink Optimization for Atrial Matrix

The first step is to develop a cardiac fibrotic bioink that mimics the composition and stiffness of fibrotic atrial tissue. This involves tuning the hydrogel formulation (e.g., collagen I and III for fibrous stiffness, hyaluronic acid for viscoelasticity) to achieve a stiffness in the range of aged/fibrotic atrium (~30–50 kPa) as opposed to healthy atrium (~5–10 kPa) [66]. By adjusting crosslinking density or blending materials, we can create a series of bioinks from “healthy soft” to “fibrotic stiff”. These will be tested for print fidelity and cell viability. Chemical cues would also be incorporated, for example, by adding Matrigel or laminin to support cardiomyocyte adhesion, and possibly peptides that modulate fibroblast behavior [67,68]. The goal is to optimize a bioink that supports both hiPSC-aCM contractility and hiPSC-CFs that are not activated [69].

### 5.2. 3D Bioprinting of Functional Atrial Tissue Constructs

Recent advances in cardiac tissue engineering have enabled the creation of 3D atrial constructs using optimized bioinks containing human-induced pluripotent stem cell-derived atrial cardiomyocytes (hiPSC-aCMs), cardiac fibroblasts (CFs), and occasionally endothelial cells to mimic the multicellular composition of native atrial myocardium [70]. Several studies have demonstrated that planar constructs, typically 10 × 10 × 1 mm^3^ in size, can be printed using aligned filament geometries such as parallel lines or interdigitated patterns. These patterns serve as contact guidance cues that promote anisotropic alignment of cardiomyocytes, improving structural and functional mimicry of atrial muscle fiber orientation [63,71].

Coaxial bioprinting strategies have also been investigated to spatially organize multiple cell types within a single fiber, such as a fibroblast-rich core surrounded by a cardiomyocyte-laden shell. This architecture has been used to simulate fibrotic niches and has demonstrated effects on electrical conduction and paracrine signaling [72,73]. Such spatial patterning enables researchers to study localized cell–cell interactions and fibrosis-mediated remodeling within a controlled 3D environment.

Following bioprinting, dynamic conditioning methods such as electrical pacing and perfusion culture have been shown to enhance cardiomyocyte maturation and electromechanical coupling significantly. For example, electrically stimulated tissues exhibit improved sarcomere organization, increased Connexin-40 (Cx40) expression—a gap junction protein expressed in atrial cardiomyocytes but not in ventricular working myocytes—and more synchronous contractile behavior [65]. Within 1–2 weeks of culture under these dynamic conditions, hiPSC-aCMs in printed constructs can form functional syncytia, exhibiting spontaneous beating and robust gap junction connectivity, which are further enhanced by the presence of fibroblasts promoting ECM deposition and maturation [74]. Together, these studies demonstrate the feasibility of using bioprinting and multi-cellular bioinks to engineer aligned, functional atrial tissues that recapitulate key physiological features and can serve as platforms for disease modeling and drug testing.

The printed tissues should exhibit an atrial-like electrophysiological profile, as demonstrated in previous studies, including action potential duration (APD) shortening in response to acetylcholine [75], rate-dependent shortening of APD with increased stimulation frequency [76], and the presence of atrial-specific ion currents such as I_Kur_ and I_KAch_ that distinguish them from ventricular phenotypes [77]. Mechanical stretch has also been shown to modulate electrophysiological properties in atrial tissues, supporting their functional relevance. Printing fidelity can enable the reproducible creation of multiple identical tissues for parallel experiments, a significant advantage for high throughput testing.

### 5.3. Controlled Fibroblast Activation (Inducing Fibrosis in Construct)

With viable atrial tissue constructs in hand, the next step would be fibroblast induction and studying fibrosis within the tissue. Rather than pre-fabricating fibrotic tissues, one approach to more accurately mimic disease progression is to induce fibroblast activation within engineered constructs over time. Fibrosis can be triggered in situ through biochemical stimuli, such as TGF-β1 or angiotensin II, or by applying mechanical cues like increased tissue stretch or substrate stiffness. By modulating the dose and duration of these stimuli, tissues with varying degrees of fibrosis—from mild interstitial changes to dense scar-like matrix—can be generated. For instance, exposure to TGF-β1 for 48 h has been shown to significantly activate human cardiac fibroblasts, with αSMA expression increasing by over 5-fold and collagen type I expression by 3-fold, accompanied by enhanced contractility and increased tissue stiffness, confirming a strong fibrotic activation response [50,78]. To ensure that fibrosis induction is specific, fibrosis-inhibiting compounds (e.g., TGF-β receptor blockers or antifibrotic agents, e.g., pirfenidone) can be included as controls. In some models, spatially restricted delivery—such as using TGF-β–soaked microbeads—has been used to create localized fibrotic regions, mimicking the patchy architecture observed in atrial fibrosis. Such strategies aim to generate engineered tissues that retain viable cardiomyocytes but experience functional alterations due to surrounding fibrosis-such as slowing electrical conduction or impaired contraction. The degree of fibrosis can then be characterized by measuring collagen content, fibroblast activation markers, and myocyte viability.

### 5.4. Fibrosis Induction and Assessment

Key soluble signals responsible for fibroblast activation include growth factors such as TGF-β and PDGFs, cytokines like IL-6 and TNF-α, and hormones such as aldosterone and Ang-II [79]. TGF-β is frequently used as an in vitro profibrotic inducer, considering it is the most important regulator of myofibroblast activation. In addition to biochemical signals, mechanical cues (such as stiffness, topography, or dynamic strain) can influence cardiac fibroblast behavior, either by maintaining their quiescent state or promoting activation to myofibroblasts. Modulating the cardiomyocyte-to-fibroblast (CM:CF) ratio further shapes the fibrotic environment; a higher proportion of CFs mimics profibrotic conditions, resulting in enhanced proliferation and activation, and collagen secretion. This can negatively affect the tissue’s electrical and contractile functions and lead to its increased ECM production and higher levels of profibrotic signals like TGF-β [38]. Most 2D models usually expose CFs to a profibrotic stimulus and assess signatures of myofibroblast activation, such as cytoskeletal reorganization and the incorporation of α-smooth muscle actin (α-SMA) into stress fibers (α-SMA, F-actin filaments, and phalloidin) [80].

Inducing a fibrotic phenotype in an engineered tissue can be achieved through several distinct methods, each mimicking a different aspect of the complex pathophysiology of human disease, as compared in Table 2. The choice of induction method is a critical experimental decision that shapes the nature of the resulting fibrotic model. For instance, acute stimulation with a high dose of TGF-β1 models a strong, rapid fibrotic response, whereas chronic mechanical stretch may better replicate the slow, progressive fibrosis associated with hypertension. This highlights a key gap and an opportunity for the field: to create more sophisticated models that combine these stimuli. Future studies could investigate how mechanical preconditioning alters the cellular response to biochemical triggers, more accurately simulating how diseases like hypertension create a vulnerable substrate that is then exacerbated by hormonal or inflammatory signals.

Marker validation is always necessary to verify the identity of reprogrammed or differentiated cells. Collagen type I, collagen type III, and fibronectin are examples of ECM structural proteins whose secretion is frequently measured. Immunofluorescence, qPCR, ELISA, Western blotting, proteomic analysis, gelatin zymography (for MMPs), or a combination of these methods are commonly used to assess the expression, localization, or activity of these markers [38].

### 5.5. Architectural and Cellular Patterning to Mimic Fibrotic Atria

To better model the structural complexity of fibrotic atria, co-culture patterning strategies can be refined to reflect tissue architectures observed in patients with atrial fibrosis. Atrial fibrosis often presents as interstitial collagen deposition around myocyte bundles, and in more advanced stages, as transmural fibrotic strands. Using bioprinting techniques, these patterns can be recapitulated by printing multicellular arrangements—for example, grids or rings of bioink with a high fibroblast-to-cardiomyocyte ratio to simulate fibrotic barriers, surrounded by regions enriched in cardiomyocytes. Composite structures such as patches with dense fibrotic cores and intact muscular peripheries may also be fabricated to mimic localized scar tissue resulting from ablation or infarction. Findings from micropatterning studies in 2D have shown that aligning fibroblasts in specific geometries can significantly influence electrical conduction and arrhythmia susceptibility. These concepts can be translated into 3D by printing linear fibrosis strands, akin to transmural septa, to evaluate their ability to induce conduction block or re-entrant circuits under rapid pacing conditions. The cellular composition can be modulated to reflect different physiological or pathological conditions, with reported myocyte-to-fibroblast ratios ranging from approximately 20:80 to 90:10. This variability can arise due to differences in species, developmental stage, tissue region, cell isolation methods, or in vitro culture conditions. Tissue constructs could be printed using varying fibroblast densities (e.g., 50% vs. 70%) to examine their effects on tissue function. Architectural fidelity may be evaluated through histological techniques, such as trichrome staining for collagen distribution, and compared to native fibrotic tissue samples. Through such strategies, engineered tissues can be designed to structurally resemble fibrotic atrial myocardium, exhibiting features such as irregular intercardiomyocyte spacing, fibroblast-rich regions, and subendocardial fibrotic layering.

The cellular composition of engineered cardiac tissue is a foundational parameter that dictates its ultimate function. However, as Table 3 illustrates, the reported ratio of cardiomyocytes to fibroblasts in the native heart is widely inconsistent, reflecting true biological variability and technical differences in measurement. This variability should not be viewed as a limitation, but rather as a critical experimental tool. The key insight for building superior models is that the cell ratio is one of the most direct ways to control the “fibrotic burden” in a dish. A model with a 90:10 CM:CF ratio will have vastly different electrophysiological and mechanical properties than one with a 50:50 ratio. Therefore, a central goal of the bioengineering roadmap is to move beyond seeking a single “correct” ratio and instead systematically modulate cell composition to investigate how different degrees of fibroblast integration impact arrhythmia triggers, conduction patterns, and therapeutic responses. This approach transforms a point of biological controversy into a powerful, titratable variable for disease modeling.

### 5.6. Pathophysiological Insights into AF and Fibrosis

Engineered atrial fibrosis models provide a unique window into studying the mechanistic interplay between fibrosis and arrhythmias. With these tissues, we can investigate how specific features of fibrosis contribute to AF, under controlled conditions that isolate variables:

#### 5.6.1. Conduction and Electrophysiology

**Figure 4:**
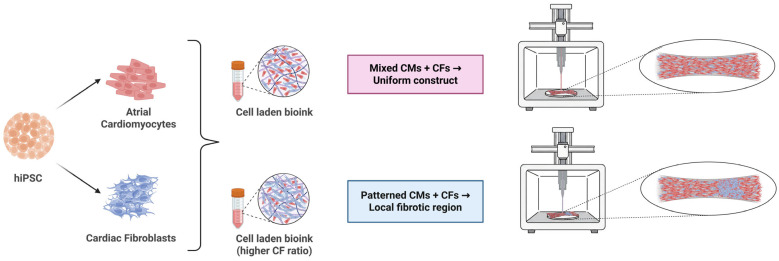
Bioprinting strategies for generating atrial tissue constructs from hiPSC-derived cardiomyocytes and fibroblasts. Human-induced pluripotent stem cells (hiPSCs) were differentiated into atrial cardiomyocytes (CMs) and cardiac fibroblasts (CFs) and incorporated into cell-laden bioink formulations. Two printing approaches were used: (i) uniform constructs generated by mixing CMs and CFs at a defined ratio prior to extrusion, producing homogeneous engineered atrial tissues; and (ii) patterned constructs in which bioinks with differential CF content were spatially deposited to create localized fibrotic regions, enabling modeling of regional fibrosis within engineered cardiac tissues. Created in BioRender. https://BioRender.com/0h040qb (accessed on 4 November 2025).

**Figure 5:**
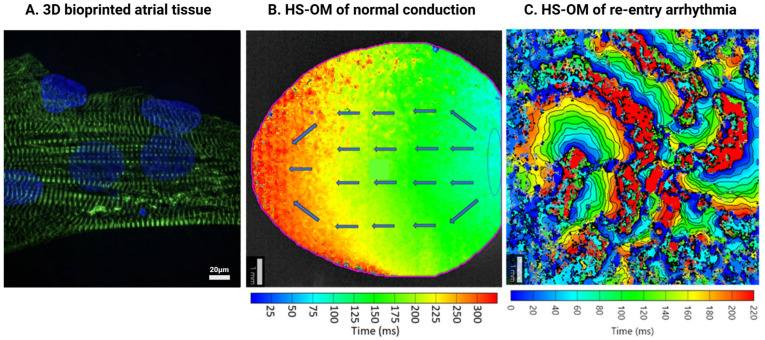
Structural and functional assessment of engineered atrial tissue. (**A**) Confocal imaging of 3D bioprinted hiPSC-derived atrial tissue showing highly organized sarcomeres and aligned cardiomyocytes. Sarcomeres are visualized via α-actinin-GFP (green). Scale bar: 20 µm. (**B**) High-speed optical mapping (HS-OM) demonstrating uniform electrical conduction across engineered atrial tissue during paced rhythm. Pixel color denotes activation time. Blue arrows indicate the direction of paced wave propagation, and the dashed marks the region of electrode-mediated stimulation. (**C**) HS-OM of re-entry arrhythmia formation in engineered tissue, displaying spiral wave activity and conduction heterogeneity.

Cardiac tissue models have become invaluable for recapitulating key aspects of human cardiac physiology and pathophysiology in vitro. Among emerging applications, the development of fibrotic cardiac tissue models has gained significant traction as a model to study cardiac fibrosis, a hallmark of many cardiovascular diseases, including heart failure and arrhythmogenic cardiomyopathies [65]. Electrophysiological studies on the bioprinted tissues can allow direct observation of impulse propagation alterations caused by fibrosis. For instance, clinical studies have shown that re-entrant circuits frequently form at the borders of fibrotic tissue [88]. Accordingly, applying multielectrode arrays (MEA) or high-speed optical mapping (HS-OM) on bioprinted tissues (Figure 4) exhibiting patchy fibrotic patterns allow quantification of key electrophysiological parameters [89] that can be adapted for high-throughput assessments. Fibrotic remodeling can disrupt normal electrophysiological function by altering ion channel expression, cellular coupling, and ECM composition (Figure 5). To understand these complex interactions at the cellular level, patch clamp electrophysiology remains a cornerstone technique for dissecting ion channel behavior in fibrotic cardiac tissue models [90].

#### 5.6.2. MEA and HS-OM Application in Fibrotic Cardiac Tissue Models

Over the past two decades, optical mapping has emerged as a pivotal technique in cardiac physiological phenotyping, offering high spatiotemporal resolution insights into the heart’s electrical activity. Electrical activity over the atrial surface can be visualized using voltage-sensitive fluorescent dyes. Its applications span from elucidating arrhythmogenic mechanisms to evaluating pharmacological interventions [91,92].

Recent studies conducted on mice have shown that AF inducibility and AF burden are enhanced in aged mice. Notably, aged mice with higher frailty index (FI) scores exhibited more sustained AF, suggesting that frailty, rather than age alone, predicts susceptibility to AF. Moreover, electrocardiographic analyses revealed that aged mice had prolonged P wave durations and PR intervals, indicative of slowed atrial and atrioventricular (AV) nodal conduction velocity. These conduction delays were strongly correlated with higher FI scores, emphasizing the impact of frailty on electrical conduction pathways. Histological assessments demonstrated increased interstitial fibrosis in the atria of aged mice. This structural remodeling was associated with alterations in ECM components and was more pronounced in mice with higher frailty scores, suggesting that frailty exacerbates age-related fibrotic changes. High temporal resolution optical mapping (>1000 fps) showed that aged and frail mice had decreased conduction velocities and shortened action potential durations in both the right and left atria. These electrophysiological changes can create a substrate conducive to re-entrant arrhythmias like AF.

Electrophysiological parameters that HS-OM can assess include conduction velocity (CV), conduction block incidence, action potential duration (APD) at different repolarization times such as APD_30_, APD_50_, and APD_90_, APD dispersion across the tissue, electrical alternans, restitution slope, and phase singularity dynamics. Each parameter can be evaluated under defined pacing protocols, including baseline pacing at 1 Hz to assess resting conduction, and progressive rate increases up to 5 Hz (300 bpm) to simulate tachycardia and unmask latent electrical instabilities. These patterned constructs can also reveal slowed conduction velocity (CV) and conduction blocks within the fibrotic regions, which is a key arrhythmogenic substrate that can lead to unidirectional block and re-entry. The disrupted cell-cell coupling around the collagen-dense regions causes the slowed CV and can be visualized by using point stimulation and isochronal mapping to visualize wavefront propagation [93,94].

The maintenance of AF is thought to be governed by two principal electrophysiological models: the multiwavelet hypothesis and the localized source (driver) hypothesis. The multiwavelet hypothesis proposes that AF persists due to the continuous formation and self-perpetuation of multiple wavelets propagating randomly throughout the atrial tissue [95]. This hypothesis postulated that, as long as the atrium had a sufficient area with adequately short refractory periods, AF could be initiated and then perpetuated indefinitely. Conversely, the “localized source hypothesis” proposes that AF is perpetuated by rapid, organized, discrete micro re-entrant circuits (rotors) or focal impulses that disorganize into fibrillatory waves at their periphery. Simulating tachycardia by pacing the fibrotic and non-fibrotic tissue constructs at high rates (e.g., 3 Hz) could induce AF-like arrhythmias in vitro [96]. At elevated pacing rates, one can analyze the onset of APD alternans, the steepness of the restitution curve, and spatial discordance of the APD to assess vulnerability to re-entrant activity. Phase mapping techniques can be used to identify and localize phase singularities and rotor activity, particularly near fibrotic borders, which allows direct assessment factors involved in generating and maintaining rotors as well as the mechanisms underlying for rotor termination by antiarrhythmic drugs used to treat AF [76]. The relationship between different fibrosis patterns and re-entrant circuits, as well as responses to ectopic burst firing, can help uncover the fibroblast-myocyte electrical coupling versus the role of conduction barriers in mechanisms whereby fibrosis can initiate and maintain arrhythmic activity.

The quantification of the minimum fibrotic burden that causes a decrease in conduction velocity can be achieved using HS-OM to identify regions where the action potential wavefront fails to propagate despite adjacent activation. Failed propagation can be found by incrementally increasing the area and density of fibrotic patches in the printed constructs and mapping propagation failure is reached [97]. This is also applicable to observe how anisotropic fibrosis affects re-entry in comparison to isotropic fibrosis. Visualizations of conduction block regions are significant as they act as anchor points for re-entrant circuits, especially around the fibrotic patches.

Pervious research shows that optical mapping techniques utilized to assess the electrophysiological properties of the engineered heart tissues during the remodeling phases. They observed that, by day 6 post-injury, the hEHTs exhibited significant electrophysiological dysfunction, characterized by reduced calcium wave conduction and activity. This decline in electrical activity indicated impaired cardiomyocyte function, a hallmark of post-myocardial infarction (MI) remodeling [98].

Despite its widespread adoption, a lack of standardized protocols for processing and analyzing optical mapping data remains a challenge. Researchers employ a range of methodologies, including image segmentation, spatial and temporal filtering, baseline drift correction, and signal normalization. The choice of processing techniques can significantly influence data interpretation, underscoring the need for consensus on optimal practices. Efforts are ongoing to refine these methodologies to enhance data reliability and reproducibility in cardiac electrophysiological studies.

MEA is often used as a complement to optic recordings. This technique measures the extracellular electric field generated when action potential propagate through the cardiac tissues. An added advantage of the MEA system is that it allows users to stimulate issues in selected regions using various temporal sequences, which can mimic programmed stimulation used clinically to identify arrhythmia vulnerability and probe the potential electrical mechanisms underlying these arrhythmias. This approach has been used recently to assess conduction velocity changes induced by fibrosis in engineered tissues [99].

Recent advances allow MEA recordings to provide high-quality, non-destructive estimates of action potential profiles in cardiac tissues. The technique is known as Local Extracellular Action Potential, or LEAP. This technique requires the application of high voltage, high frequency electrical signals in extracellular microelectrodes to create strong cell-to-cell coupling, allowing assessment of AP morphology such as rise times and action potential durations, which are useful for evaluating drug effects or genetic modifications on cardiac electrophysiology. A notable application of LEAP technology was the use of this technique for identifying different cardiomyocyte subtypes in engineered patches [100]. LEAP measurements have been used to assess the effects of hypoxia on AP profiles in monolayer tissues [101].

#### 5.6.3. Assessment in Fibrotic Cardiac Tissue Models Using Patch-Clamp Measurements

Patch clamp electrophysiology, especially in the whole-cell configuration, is crucial for interrogating the electrophysiological phenotype of cardiomyocytes within fibrotic constructs. Given the technical difficulty of directly accessing embedded cells within 3D tissue, dissociation of fibrotic cardiac tissue models into single cells or microdissection of thin tissue sections is often employed prior to recording [28].

Beyond cardiomyocytes, patch-clamp techniques are increasingly used to characterize the electrophysiology of cardiac fibroblasts themselves. Fibroblast ion channels—including transient receptor potential (TRP) channels, K^+^ channels, and STIM/Orai channels—are now recognized as critical regulators of pro-fibrotic signaling and gene expression. The activity of these channels contributes to processes such as calcium-dependent transcription factor activation and “excitation-secretion coupling,” a mechanism where ion fluxes trigger the release of collagen and other ECM proteins, directly contributing to tissue remodeling [102].

AF is closely associated with both electrical and structural remodeling of the atria, each potentially contributing to the initiation and maintenance of the arrhythmia. Electrical remodeling typically involves alterations in ion channel expression and function, leading to changes in action potential (AP) morphology. Specifically, the sodium current (I_Na_), mediated by Na_V1.5_ channels, is crucial for the rapid upstroke of the AP and efficient impulse propagation across the atrial myocardium. The duration of the AP is determined by a balance between the inward L-type calcium current (I_Ca,L_), carried by Ca_V1.2_ and Ca_V1.3_ channels, and various repolarizing potassium currents, including the transient outward current (I_to_), and the ultrarapid delayed rectifier current (I_Kur_). In species such as humans, dogs, and rabbits, additional currents like the rapid (I_Kr_) and slow (I_Ks_) delayed rectifier potassium currents are also significant determinants of action potential profile and duration. Repolarizing delayed rectifier K^+^ currents generated by I_Kr_ and I_Ks_, as well as the inward rectifier K^+^ current (I_K1_), are particularly critical for providing the appropriate levels of repolarization reserve needed in the late stages of repolarization to overcome the arrhythmogenic consequences of Ca^2+^ current reactivation. Collectively, alterations in either repolarizing or depolarizing currents can lead to changes in AP profile and duration that promote arrhythmias; for instance, AP prolongation can increase the risk of early afterdepolarizations (EADs) and conduction block, while conversely, AP shortening can favor re-entry by reducing the wavelength of the propagating impulse. Furthermore, arrhythmogenic triggers can also arise from delayed afterdepolarizations (DADs), which are typically caused by enhanced sarcoplasmic reticulum (SR) Ca^2+^ release and the resulting depolarizing current via the sodium-calcium exchanger (NCX) [103,104].

Fibrotic remodeling is associated with ion channel expression changes that mimic pathological remodeling seen in human heart failure. For instance, studies using fibrotic EHT have reported decreased expression of *KCNJ2* (coding for Kir2.1), a molecular component of I_K1_, and *SCN5A* (encoding Nav1.5 underlying I_Na_), consistent with the changes in these currents seen in failing myocardium. These changes are known to compromise the cardiac action potential’s stability and conduction safety factor [105].

Pharmacological studies using patch clamp have provided insights into the proarrhythmic substrate in fibrotic environments. For example, the selective blockade of repolarizing potassium currents (e.g., IKr or IKs) in fibrotic cardiac tissue models results in exaggerated APD prolongation compared to non-fibrotic controls, suggesting a reduced repolarization reserve in fibrotic tissues. Additionally, SK channel blockers like apamin have been used to explore the role of small-conductance calcium-activated potassium channels (e.g., *KCNN2*, *KCNN3*), which may become upregulated or downregulated in response to calcium overload and fibrosis-induced signaling cascades [106].

The incorporation of fibrotic features into engineered heart tissue enhances its physiological relevance for modeling human cardiac disease. Patch clamp electrophysiology plays a pivotal role in this context, offering high-resolution insights into ion channel remodeling, altered excitability, and arrhythmia susceptibility in fibrotic environments. By combining cellular electrophysiology with fibrotic tissue modeling, researchers can better understand the mechanisms by which fibrosis contributes to cardiac electrical dysfunction and test targeted antiarrhythmic or antifibrotic therapies in a human-relevant system.

Cardiomyocytes from fibrotic cardiac tissue models typically exhibit prolonged action potential duration (APD), depolarized resting membrane potential (RMP), and reduced upstroke velocity—all indicative of altered ionic current dynamics. Patch clamp recordings have shown that fibrosis is associated with downregulation of inward rectifier potassium current (I_K1_), contributing to a destabilized RMP and increased automaticity. Moreover, reductions in sodium current (I_Na_) density and impaired sodium channel kinetics can slow conduction and predispose tissue to re-entrant arrhythmias [104].

Studies have also demonstrated altered calcium handling in fibrotic models. L-type calcium current (I_Ca,L_) may be reduced, while calcium-dependent delayed afterdepolarizations (DADs) can emerge more frequently due to disrupted excitation–contraction coupling and increased intracellular calcium variability. In some fibrotic cardiac tissue models, fibroblasts themselves may form gap junctions with cardiomyocytes, contributing to electrotonic loading and further modifying AP morphology, though this remains an area of active investigation [88].

#### 5.6.4. Fibroblast-Cardiomyocyte Crosstalk

The co-culture nature of the model allows dissection of electrical coupling between fibroblasts and cardiomyocytes. Fibroblasts in the heart can influence excitability by electrotonic coupling (through gap junctions) and by secreting paracrine factors. In this model, fibroblast coupling can shorten cardiomyocyte action potentials or slow their upstroke, as has been hypothesized in fibrotic hearts. These experiments address questions like: do fibroblasts primarily act as passive insulators, or active electrical participants in the AF substrate? Early evidence suggests that fibroblast connexins (Cx43, Cx40) and their associated Na^+^ currents modulate conduction; this model provides a means to test this by using fibroblasts with specific ion channel knockouts.

#### 5.6.5. Characterizing Fibrotic Features Using ICC, Flow Cytometry, and RNA-Seq

Once the fibrotic model is established and demonstrates distinct features compared to healthy controls, the next step is to characterize how different culture conditions, such as external stimuli or genetic variants, affect the development of the fibrotic environment. This involves readout parameters that largely overlap with those used during model characterization, such as the expression of matrix remodeling genes (collagens, assembly proteins, and proteases) using RNA sequencing, and mesenchymal markers (α-SMA, fibronectin, and vimentin) using immunocytochemistry and flow cytometry [38].

Figure 4 shows the progressive transition from quiescent fibroblasts to proto-myofibroblasts and fully differentiated myofibroblasts. Under mechanical tension, fibroblasts form stress fibers and focal adhesions, becoming proto-myofibroblasts. With sustained mechanical tension, TGF-β1 signaling, and deposition of ED-A fibronectin, these cells further differentiate into myofibroblasts characterized by abundant α-smooth muscle actin (α-SMA) stress fibers and enhanced contractility. Myofibroblasts can potentially revert to fibroblasts under certain conditions, though this reversibility remains incompletely understood. Confirming a fibrotic phenotype in an engineered tissue requires more than just observing an increase in collagen. A robust validation framework must employ a multi-modal assessment strategy to connect changes across molecular, cellular, and functional levels. A critical challenge in the field is the over-reliance on a limited set of markers, such as α-SMA, which is not exclusively expressed by myofibroblasts and can be present in other cell types. Therefore, a key takeaway is that no single marker or technique is sufficient. A truly validated model of atrial fibrosis must demonstrate not only the activation of fibroblasts and deposition of ECM but also the direct functional consequences of these changes. Correlating these multi-scale measurements is essential for building a complete and convincing picture of the disease state in a dish [107].

Comprehensive characterization begins at the cellular level by confirming the activation of quiescent fibroblasts into myofibroblasts. This transition can be verified by assessing key markers like α-Smooth Muscle Actin (ACTA2) and Periostin (POSTN) using a combination of techniques: immunocytochemistry provides spatial localization, flow cytometry allows for quantitative analysis of cell populations, and RT-qPCR or RNA-Seq measures changes in gene expression. The next step is to quantify the structural hallmark of fibrosis—the excessive deposition and remodeling of the extracellular matrix. The increased presence of key proteins like Collagen I (COL1A1), Collagen III, and Fibronectin can be confirmed through histological staining (e.g., Trichrome), ELISA, and proteomic analyses. Ultimately, these cellular and structural alterations must be linked to the electrophysiological remodeling that drives arrhythmias. This involves measuring changes in the expression of genes encoding connexins (e.g., Cx40) and ion channels (e.g., KCNJ2, SCN5A) and directly assessing their functional impact—such as slowed conduction or altered action potentials—with high-speed optical mapping and patch clamp electrophysiology [108,109].

#### 5.6.6. RNA Extraction, Reverse Transcription, and Real-Time Quantitative PCR Analysis

RNA extraction from cultured cardiac cells followed by reverse transcription into complementary DNA (cDNA) and subsequent quantitative PCR (RT-qPCR) is a common approach to quantify gene expression changes at the bulk tissue or mixed cell population level. For example, Niro et al. successfully utilized this method in induced pluripotent stem cell-derived cardiac fibrosis models to assess transcriptional alterations associated with fibrotic remodeling [41]. However, in co-culture systems where multiple cell types are present, bulk RNA analyses present a critical limitation: they cannot distinguish which specific cell types are responsible for the observed gene expression changes. This lack of resolution complicates the interpretation of results, as gene expression signals are averaged across all cells in the sample. To address this challenge, researchers increasingly rely on advanced transcriptomic technologies such as single-cell RNA sequencing (scRNA-seq), single-nucleus RNA sequencing (snRNA-seq). Together, these methods make it possible to study gene expression in detail and clearly identify the different cell types within mixed co-cultures or tissue models, helping us better understand how each cell type contributes to cardiac fibrosis. These approaches enable profiling of gene expression at the resolution of individual cells, thereby allowing precise identification of cell type-specific transcriptional signatures within heterogeneous co-cultures or tissue models. This significantly enhances the ability to understand the contributions of different cell populations in cardiac fibrosis [110].

Alternatively, physical isolation of individual cell types prior to RNA extraction—using methods such as fluorescence-activated cell sorting (FACS)-can be performed to enable cell type-specific gene expression analyses. This approach, while more labor-intensive, provides a targeted strategy to overcome the cell type ambiguity inherent in bulk analyses [41].

#### 5.6.7. Flow Cytometry

Flow cytometry can be used to quantify changes in specific cell populations and marker expression before and after fibrosis induction. This method allows for the detection of both surface and intracellular proteins at the single-cell level, making it valuable for analyzing mixed cultures such as atrial cardiomyocytes (hiPSC-aCMs) and cardiac fibroblasts (hiPSC-CFs).

In this model, flow cytometry can be used to assess the proportion of fibroblasts versus cardiomyocytes by staining for markers such as DDR2 and Vimentin (fibroblast marker), cTnT (cardiomyocyte marker), and MLC2a (atrial cardiomyocyte marker). To evaluate fibrosis induction, additional markers like α-SMA (myofibroblast activation) and FAP (activated fibroblasts) can be included. Changes in marker expression levels between treated and control samples provide quantitative insights into cell activation, phenotype shifts, and potential fibrotic remodeling [41]. For instance, a study by Biendarra-Tiegs et al. [111] confirmed efficient separation of cardiomyocytes and non-cardiomyocytes using cTnT and CD90 markers, allowing precise monitoring of fibroblast-like cells over time in co-culture setting. This method enables the simultaneous multiplexing of multiple markers, facilitating the development of a comprehensive understanding of cell populations and their responses to fibrotic stimuli. It also complements insights from ICC and the transcriptomic depth provided by RNA-seq.

#### 5.6.8. Immunocytochemistry (ICC)

Immunocytochemistry can be used to visualize and localize key fibrosis-related proteins in co-culture models of hiPSC-aCMs and hiPSC-CFs. This technique allows for the assessment of structural and morphological changes that occur during fibrosis induction. Cultured model atrial tissues should be fixed and permeabilized with 4% paraformaldehyde and 0.1% Triton X respectively. The preparations can then be labeled for markers such as α-SMA (for myofibroblast), FAP (for activated fibroblast), fibronectin, and collagen I (for ECM deposition) can be stained to confirm fibroblast activation and fibrosis development. For cardiomyocytes, markers like cTnT, MLC2a, and K_V_1.5 (atrial-specific ion channels) are used to identify and assess atrial cell structure and organization [37]. Images can be acquired using a confocal microscope, which allows for high-resolution imaging of fluorescently labeled cellular structures in multiple focal planes (z-stacks). The acquired images can then be processed and analyzed using ImageJ (or Fiji) version 1.54r, an open-source image analysis software [37]. This provides visual confirmation of cellular changes that complement the quantitative data obtained from flow cytometry and RNA-seq.

#### 5.6.9. Matrix Biology in AF

Engineered heart tissues offer a valuable platform for studying fibrotic remodeling in AF, as they allow for controlled manipulation and biochemical analysis of tissue responses. Studies using rapid electrical pacing of EHTs have mimicked persistent AF conditions, revealing changes in ECM protein expression, such as increased collagen levels, which are indicative of fibrotic remodeling [109,112]. These in vitro findings parallel observations from animal models, where chronic AF leads to progressive atrial fibrosis over time. Importantly, the use of patient-specific hiPSC-derived cardiomyocytes enables investigation of inter-individual variability in fibrotic responses, reflecting clinical evidence that fibrotic burden differs among AF patients. In some cases, hiPSC-derived models have demonstrated predisposition to fibrosis under stress, highlighting their potential for personalized disease modeling [113].

Moreover, insights from these models can inform therapeutic strategies; for instance, studies have shown that blocking fibroblast–cardiomyocyte coupling may reduce arrhythmic activity, supporting the exploration of fibroblast connexins or adhesion molecules as drug targets. These platforms also facilitate mechanistic investigations of genotype–phenotype relationships. For example, introducing AF-associated genetic variants such as titin truncations into hiPSCs and differentiating them into atrial tissue can reveal how specific genetic variants contribute to fibrotic remodeling and arrhythmogenic behavior, thus bridging the gap between genetic discoveries and functional outcomes [76].

#### 5.6.10. Ca^2+^ Handling

Calcium handling abnormalities are a hallmark of AF, including phenomena such as spontaneous calcium release, delayed afterdepolarizations, and calcium alternans, which can lead to ectopic activity and arrhythmogenesis. At the molecular level, these are often driven by key protein dysfunctions, such as the hyper-phosphorylation of the ryanodine receptor (RyR2) leading to diastolic Ca^2+^ leak, and the downregulation or dysfunction of the sarcoplasmic/reticulum Ca^2+^-ATPase (SERCA), which impairs Ca^2+^ reuptake [114]. In engineered tissue models, calcium transients can be evaluated using fluorescent calcium indicators (e.g., Calbryte 630, Fluo-4), enabling the visualization and quantification of calcium wave propagation across fibrotic and non-fibrotic regions. These Ca^2+^ reporters give a sense of relative, not absolute, cytosolic Ca^2+^ level changes. For a more absolute measurement, the ratiometric Ca^2+^ reporter Fura-2 (excitation 340 nm/380 nm) can be used with a known K_d_ for Ca^2+^ under specific conditions. Disruptions in calcium signaling, such as delayed propagation, alternans, or regional desynchrony, may emerge in tissues containing dense fibroblast populations or irregular ECM deposition, mimicking patient-specific calcium dysregulation patterns seen in AF.

In parallel, sarcomere organization serves as a key indicator of cardiomyocyte structural maturation and contractile competence, which is critical for understanding the atrial myopathy associated with AF. Immunofluorescence imaging targeting sarcomeric α-actinin or titin can be used to quantify sarcomere alignment, length, and striation clarity. Prior studies have shown that co-culturing cardiomyocytes with supportive stromal cells, including fibroblasts, may enhance sarcomere organization under optimized conditions. However, excessive fibroblast density or ECM stiffness may impair contractile alignment. To assess this functionally, automated sarcomere tracking algorithms can be applied to high-frame-rate contraction videos to measure sarcomere shortening, which reflects the amplitude of cardiomyocyte contraction and serves as a direct index of tissue-level contractility, a key parameter that declines during AF progression.

In fibrotic constructs, mechanical constraint imposed by activated fibroblasts or collagen accumulation may lead to reductions in sarcomere shortening or delayed relaxation, indicating impaired excitation–contraction coupling. Together, calcium imaging and sarcomere analysis offer complementary insights into how fibrosis affects the intracellular machinery and contractile performance of atrial cardiomyocytes, forming a critical link between tissue remodeling and the functional deterioration characteristic of AF.

#### 5.6.11. Mitochondrial and Metabolic Assessment

AF and fibrosis can both impact the metabolic state of atrial cells. Analyzing mitochondrial function in tissues can be achieved by measuring oxygen consumption rate (OCR) and extracellular acidification (via Seahorse assays) for small tissue pieces, or by using micro-O_2_ sensors in the culture. Atrial myocytes under tachycardic stress may exhibit reduced oxidative capacity (OCR) or a shift toward glycolysis; fibrosis may further impede oxygen diffusion. Fluorescent probes can be used to measure mitochondrial membrane potential (e.g., TMRM) and determine if myocytes in fibrotic regions are under higher oxidative stress. Given that fibroblasts can sequester fuel or produce cytokines, there might be metabolic remodeling in co-culture. For instance, a recent study found that enhanced ketone metabolism alleviated stress in fibrotic atria—it can be tested to determine metabolic modulators on the tissues and immediately measure the effect on beat rate or contractility.

#### 5.6.12. Validation Against Native Tissue

As a form of benchmarking, comparison can be made between engineered tissues and actual human atrial tissue samples (e.g., from surgery) in terms of key metrics, including stiffness, collagen fraction, gene expression profile, and electrophysiological properties. This helps ensure that the model is representative of the real disease condition. If discrepancies exist (for example, if the model has higher pacemaker activity than real atria), we can adjust parameters, such as adding atrial-specific fibroblast subtypes or nervous innervation in future iterations.

## 6. Future Perspectives and Conclusions

Engineering fibrotic atrial tissue in vitro by integrating hiPSC technology, 3D bioprinting, and careful biomaterial design stands to transform AF research and therapy development. This review outlined a conceptual roadmap for building such models and highlighted the rationale that motivates each step. Looking ahead, several emerging avenues and implications warrant further explanation.

### 6.1. Applications of Engineered Fibrotic Atrial Tissue Models

Engineered fibrotic atrial tissues provide a powerful in vitro platform for modeling AF by enabling precise control over cellular composition, electrical pacing, and biochemical environment in a physiologically relevant, human-specific context. These models facilitate high-throughput drug screening by allowing patient-specific miniaturized tissues to be exposed to candidate compounds, with functional readouts such as contraction amplitude, conduction velocity, and arrhythmia susceptibility measured via calcium imaging or electrode arrays. This approach helps identify drugs that target fibrosis and electrical remodeling simultaneously, improving translational relevance. Furthermore, engineered tissues support testing of anti-fibrotic therapies, including pharmacologic agents and gene therapies, under chronic dosing regimens to assess efficacy, toxicity, and reversal of fibrosis-related markers and functional impairments.

Patient-derived induced pluripotent stem cells enable the 3D bioprinting of atrial tissues that replicate individual genetic backgrounds and fibrotic phenotypes, providing a foundation for personalized medicine. These personalized tissue models support ex vivo screening of drugs to determine patient-specific responses, guiding tailored therapeutic strategies. While these models hold promise for more comprehensive disease modeling—including the impact of comorbidities like heart failure or metabolic disorders—these applications remain speculative and are better positioned as future directions in conclusive discussions. Ultimately, engineered fibrotic atrial tissues represent a versatile and translational platform to elucidate the mechanistic underpinnings of AF and accelerate the development of targeted, individualized therapies.

### 6.2. Personalized Cardiac Models

This approach is well aligned with the goals of personalized medicine. By leveraging patient-derived hiPSCs, researchers can generate atrial tissue models that reflect individual genetic background and comorbidities. These personalized constructs enable “clinical trials in a dish” where multiple therapies are tested on a patient’s own tissue analog before determining the most effective course of treatment. As hiPSC derivation becomes increasingly streamlined and accessible, it is feasible that clinicians could rely on these models to decide between a new antifibrotic drug, a gene therapy, or proceed to ablation for a patient with therapy-resistant AF. Moreover, biobanks of bioprinted tissues from genetically diverse patients can help us understand population variability in disease presentation, helping to explain why some patients exhibit extensive fibrosis with minimal arrhythmia, while others develop AF in the absence of overt fibrotic remodeling. Such insights are critical to advancing precision cardiology and improving patient-specific care.

### 6.3. Integration of Additional Cell Types and Complexity

While this study’s focus is on atrial CMs and fibroblasts, future iterations can incorporate other cell types relevant to the atrial substrate. For example, endothelial cells (from hiPSCs) could be printed to form capillaries within the tissue, improving cell survival and adding the endocardial interface that might modulate fibrosis (endothelial-to-mesenchymal transition could even generate fibroblasts in situ). Immune cells like macrophages could be introduced to study their role in post-injury fibrosis and inflammation-driven AF. Ultimately, one could envision a mini-atrial chamber model with a lumen, endocardium, myocardium, and epicardial fat—each contributing to AF (epicardial adipose tissue and fibro-fatty infiltration are factors in AF for some patients). 3D bioprinting is adaptable to building such multilayered structures, especially as resolution and multi-material printers’ advance.

### 6.4. Maturation and Scaling

Advances in tissue maturation strategies, including electromechanical stimulation and integration with organ-on-chip microfluidic platforms, are expected further to enhance the physiological accuracy of engineered atrial tissues, potentially enabling the achievement of adult-like conduction velocities and contractile forces. Scaling these tissues to centimeter-scale dimensions could support the induction and maintenance of sustained re-entrant AF-like activity in vitro, providing a valuable platform for direct visualization of arrhythmic circuits and testing of interventional strategies such as ablation or defibrillation. Conversely, miniaturization into high-throughput formats, such as 96-well plates containing microtissues, offers a scalable solution for efficient drug screening. This approach can bridge the gap between conventional cell-based assays and resource-intensive animal models, enabling parallel testing of candidate compounds in a physiologically relevant, human-specific context.

### 6.5. Therapeutic Development

The knowledge gained from these models can drive the development of novel therapies. For example, if the model confirms that fibroblast-cardiomyocyte coupling via Cx43 is pro-arrhythmic, one might develop a targeted gap junction modulator that uncouples fibroblasts without affecting myocyte-myocyte coupling. If certain microRNAs emerge as master regulators of atrial fibrosis (as suggested by prior studies), patient-specific tissues can test microRNA therapies in a preclinical human platform. Additionally, bioprinted patches of healthy atrial tissue might one day be used as implants to replace fibrotic regions or as biologically paced patches to restore atrial function—an intersection of regenerative medicine and bioengineering.

### 6.6. Challenges and Next Steps

Despite recent progress, several key challenges remain. Achieving full adult-like maturity in hiPSC-derived atrial cardiomyocytes continues to be a major objective, particularly with respect to atrial-specific features such as proper Connexin 40 expression and presence of mature atrial granules that contain and secrete atrial and brain (B-type) natriuretic peptides (ANP/BNP). Fibroblast heterogeneity is another challenge since not only do atrial fibroblasts differ from their ventricular counterparts, but there are also distinct atrial subsets (e.g., appendage vs. septum). Incorporating this cellular diversity will be important for ensuring biological fidelity. Moreover, long-term stability of printed tissues needs to be validated if we are to model chronic AF conditions and ensuring reproducibility between batches and printers will be important for widespread adoption.

In addition, vascularization remains a major challenge for the viability and functional integration of thicker, bioprinted tissues. The absence of perfusable microvasculature limits nutrient and oxygen transport, particularly in larger or long duration constructs, which compromise tissue viability and function. However, recent efforts have enabled significant progress, particularly in cardiac organoid models where the co-differentiation of endothelial cells has led to functional vascularization. This drastically improves the translatability of these models for studying disease development and progression [115]. Efforts to integrate these principles into larger engineered constructs, either through co-printing strategies, angiogenic stimulation, or bioreactor-based perfusion systems, are crucial for achieving more physiologically relevant models. Finally, incorporation of innervation, immune components, and mechanical stimulation may be necessary for fully recapitulating the atrial niche. Despite these, the trajectory of innovation is clear: with interdisciplinary collaboration, these bioengineered atrial models will continually improve.

In conclusion, the convergence of stem cell biology, 3D bioprinting, and tissue engineering is enabling the creation of ECM-integrated cardiac models that were not possible just a few years ago. By examining the role of atrial fibrosis in AF through these models, we can unravel disease mechanisms in a human-relevant context and open new frontiers for treatment. This approach exemplifies a shift from reactive treatment of AF to a proactive understanding of the patient’s cardiac landscape, ultimately striving to reduce the burden of AF through tailored, mechanism-guided interventions. The promise of patient-specific, bioprinted atrial tissues is that one day, we may predict and prevent an individual’s AF progression by studying a piece of their heart tissue in the lab—a prime example of technology bringing us closer to truly personalized medicine for cardiovascular disease.

## Figures and Tables

**Table 1:** Comparison of Atrial Fibrillation (AF) Models.

Model Type	Advantages	Limitations	Key Applications	Key References
**Animal Models (e.g., mouse, canine, porcine)**	High physiological relevance; captures systemic, neurohumoral, and immune factors. Enables study of complex 3D arrhythmia patterns in an intact heart.	Species differences in electrophysiology (ion channels, heart rate) limit human translatability. High cost and low throughput, especially for large animals.	Studying systemic contributors to AF and fibrosis progression.	[1,9,19,20]
**Ex Vivo Tissue Slices (e.g., human atrial trabeculae)**	Preserves native 3D architecture, cell types, and ECM. Directly uses human tissue, enhancing translational relevance.	Limited viability (days to weeks), preventing long-term studies. High patient-to-patient variability and difficulty obtaining healthy controls.	Acute drug testing and electrophysiological studies on intact human tissue.	[21,22,23,24,25,26]
**2D Monolayer Cell Culture**	Low cost, high-throughput, and easy to analyze. Precise experimental control over cell composition and environment.	Lacks 3D architecture and native ECM, leading to unnatural cell behavior and fibroblast activation. Primary cells rapidly de-differentiate.	High-throughput screening and basic mechanistic studies of single cells.	[37,38,39,40,41]
**3D Bioprinted Tissues**	High control over 3D architecture, cell composition, and spatial patterning to mimic fibrotic heterogeneity. Fully human system using patient-specific hiPSCs, avoiding species mismatch. Enables long-term culture with enhanced maturation.	Technically complex and can have lower throughput than 2D models. Achieving full adult-like maturity and vascularization remains a challenge. Protocols are lengthy, costly, and labor-intensive, from initial differentiation to the long-term culture (weeks to months) required for maturation.	Patient-specific disease modeling, mechanistic studies of fibrosis–arrhythmia links, and preclinical therapeutic testing.	[38,42]

**Table 2:** Methods for Inducing Fibrosis in Engineered Cardiac Models.

Induction Method	Mechanism	Advantages	Limitations
**Biochemical (e.g., TGF-β, Ang-II)**	Soluble factors like growth factors, cytokines, and hormones activate intracellular signaling cascades that promote myofibroblast activation and collagen synthesis.	Highly potent and reproducible; TGF-β is considered the most important regulator of myofibroblast activation. Easy to implement in culture and directly mimics key pathological signaling pathways.	May not fully capture the chronic, low-grade inflammation or mechanical stress that drives fibrosis in vivo.
**Mechanical (e.g., substrate stiffness, cyclic stretch)**	Fibroblasts sense and respond to mechanical cues like stiffness, topography, or dynamic strain, which can promote their activation to myofibroblasts.	More closely mimics the mechanical overload conditions seen in hypertension or heart failure. Can be applied dynamically to study mechanotransduction.	Requires specialized equipment like stretchers or tunable hydrogels. Can be harder to standardize across experiments compared to biochemical stimuli.
**Cellular (e.g., increasing CF:CM ratio)**	A higher proportion of cardiac fibroblasts (CFs) mimics profibrotic conditions, resulting in their enhanced proliferation, activation, and secretion of collagen and pro-fibrotic signals like TGF-β.	Simple to implement during the initial tissue construction phase. Directly models the increased fibroblast cell number observed in some diseased tissues.	Does not model the activation of resident fibroblasts in response to a stimulus but rather starts with a pre-determined pro-fibrotic cell density.

**Table 3:** Cardiomyocyte-to-Fibroblast Ratios in Native Tissue and In Vitro Models.

Context	Reported Cardiomyocyte (CM): Fibroblast (CF) Ratio	Implication/Rationale for Modeling	Citation
**Native Human Heart Tissue**	Highly variable and controversial; estimates range from 1:10 to 4:1 (CM:CF). Varies by species, cardiac region, and measurement technique.	The lack of a single “correct” ratio in native tissue means that in vitro models should treat the ratio as a key experimental variable to be systematically tested, rather than a fixed parameter.	[81,82,83]
**In Vitro “Healthy” Model**	Commonly ~3:1 to 4:1 (CM:CF).	This ratio mimics a myocyte-rich environment while still including fibroblasts to support cardiomyocyte maturation, ECM production, and electrical function.	[84,85]
**In Vitro “Fibrotic” Model**	Commonly inverted to ~1:3 (CM:CF) or uses even higher fibroblast densities (e.g., ~70% CFs).	A higher proportion of fibroblasts simulates profibrotic conditions, promoting excessive ECM deposition, increased tissue stiffness, and altered electrophysiology characteristic of disease.	[41,86,87]

## Data Availability

The original contributions presented in this study are included in the article. Further inquiries can be directed to the corresponding author.
